# Three Trocars Laparoscopic Resection of Angiomyolipoma of the Liver

**DOI:** 10.4061/2011/150691

**Published:** 2011-03-14

**Authors:** J. M. Ramia, R. De la Plaza, J. Quiñones, M. D. Sanchez-Tembleque, A. Caminoa, P. Veguillas, J. García Parreño

**Affiliations:** ^1^Hepatopancreatobiliary Unit, Department of Surgery, Guadalajara University Hospital, 19002 Guadalajara, Spain; ^2^Department of Gastroenterology and Hepatology, Guadalajara University Hospital, 19002 Guadalajara, Spain; ^3^Department of Pathology, Guadalajara University Hospital, 19002 Guadalajara, Spain

## Abstract

Angiomyolipoma of the liver (AML) is an infrequent neoplasm composed of three tissues (adipose, muscle and vessels). In spite of advances in radiology, preoperative correct diagnosis is difficult. Clasically, a conservative management strategy was adopted in patients with asymptomatic tumors less than 5 cm with undoubtful diagnosis. But after publishing some few cases of malignant angiomyolipoma a more radical has been advocated. Laparoscopic resection of liver tumors is becoming a excellent approach for operating on benign liver tumors. Usually is performed using five trocars but in some cases a less invasive technique with three trocars could be used. We present a laparoscopic resection of liver angiomyolipoma in a 65 year-old male using only three trocars and also discuss the optimal management of AML and technical tips of three-trocar technique.

## 1. Introduction

The angiomyolipoma of the liver (AML) is a very infrequent benign tumor characterized by three components: adipose, vascular, and muscular [1–7]. Progresses achieved in imaging techniques have substantially increased the number of correct preoperative diagnoses of AML [5]. The publications in the medical literature of some cases that confirm a malign transformation of AML have made the surgical indications for this pathology a controversial issue [3, 4, 6]. Laparoscopic approach, when feasible, is a technique of choice that is gaining ground and which is commonly performed with the use of 4 or 5 trocars [8, 9]. We describe here a new case concerning a laparoscopic resection with only three trocars; we have revised the literature and discussed surgical indications.

## 2. Case Report

**Figure 1:**
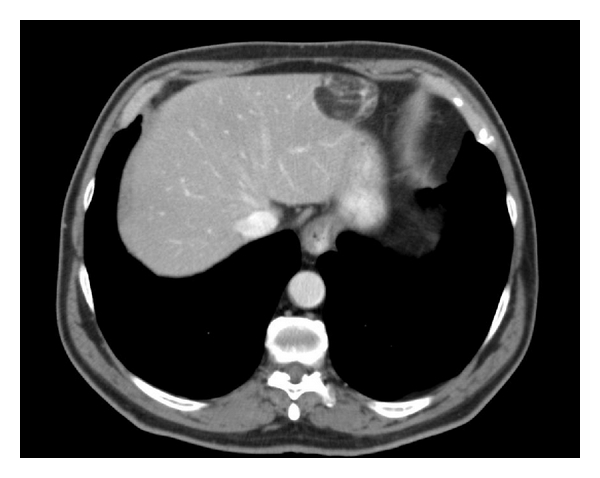
Abdominal CT showing a peripheral lipomatous lesion in left lateral sector.

**Figure 2:**
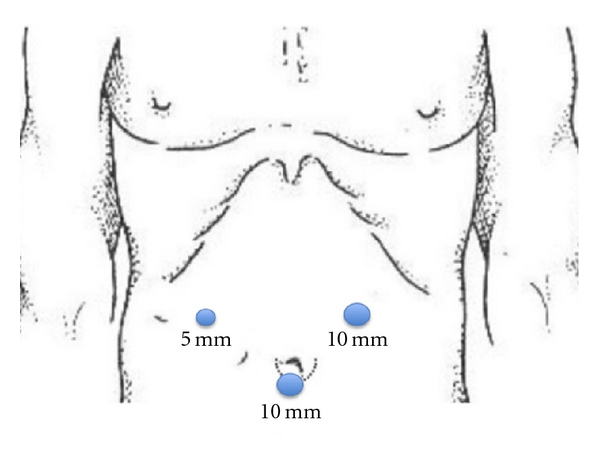
Trocar placement.

**Figure 3:**
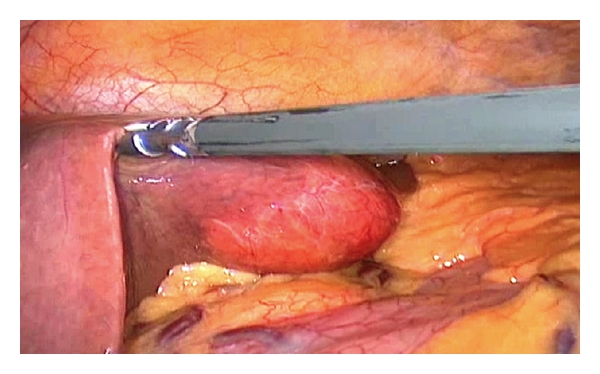
Intraoperative image of liver resection.

**Figure 4:**
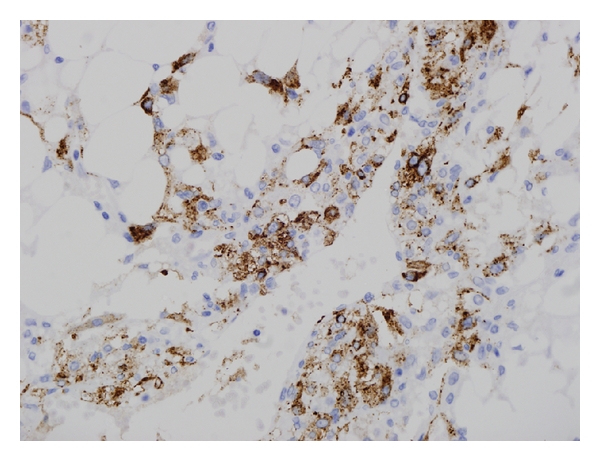
Typical HMB45 staining of liver angiomyolipoma.

A 65-year-old male patient presented with epigastric pain, with no medical records of interest, and was not affected by tuberous sclerosis. Ultrasonography and abdominal CT (Figure 1) revealed an adipose-looking lesion of around 4 cm located on the left lateral hepatic sector. Laboratory findings and tumoral markers were unremarkable. On account of the symptomatic AML diagnosis, it was decided to perform surgical resection of the lesion. The patient was placed in the French position: the surgeon between his legs and the assistant on the left side of the patient. Three trocars were placed: a 10 mm trocar in umbilical position for the 30° camera, a 5 mm trocar placed in the right hypochondrium, and another 10 mm trocar (operator's trocar) in the left hypochondrium (see Figure 2). Pringle's maneuver was prepared passing a nylon loop, although no vascular control was applied. A partial left sectionectomy was carried out, sealing the parenchyma with Ligasure 5 mm (Figure 3). The piece was removed in a bag through the umbilical trocar, widening the incision up to 2 cm. The procedure lasted 40 minutes, and the loss of blood amounted to 75 cc. The patient did not show morbidity. The in-hospital stay was three days. Macroscopically, it is a well-delimited 4 cm lesion, although not encapsulated. The margin between AML and surgical cut was 6 mm. The histological study revealed a mesenchymal lesion with a muscular, adipose, and vascular component. The muscular component showed epithelioid cells. Immunohistochemical study was negative for pankeratin, AE1-AE3, and CD117 but positive for S-100 with reference to the adipose tissue; HMB45 was positive within the cytoplasm of spindle-shaped and epithelioid cells and also for actin and desmin in the vascular component of the thick wall (Figure 4), so the final diagnosis confirmed a benign mixed angiomyolipoma. Any atypical cell was seen in every component of AML and p53 was negative. The radiological control carried out 12 months later was normal and did not show any relapse, and the patient is now free of symptoms.

## 3. Discussion

Regularly, the hepatic angiomyolipoma (AML) is a solitary mesenchymal tumor, not encapsulated and of a variable size (1 to 36 cm) [1, 5, 6] that is composed of three variable tissues: muscular cells, thick-wall vessels, and mature adipose tissue [1–7]. It occurs more frequently in women, with no age preference, and has a low incidence. It was described for the first time by Ishak in 1976, and there are only 300 cases published ever since [1–6]. Commonly, it is a sporadic tumor, but it is associated with tuberous sclerosis in 6% of patients [1, 2]. This predisposition suggests that genes involved in the tuberous sclerosis (TSC1 and TSC2) may be instrumental in AML pathogenesis [7]. AML muscular cells are called PEC (perivascular epithelioid cell), while identical cells are seen in other tumors (lung lymphangiomatosis, lung or pancreas clear cells, cardiac rhabdomyoma, etc.), so it has been suggested to include these tumors in a family called PEComas, although this idea is not widely accepted [1, 2]. 

There are 3 types of AML, histologically speaking, depending on the amount of fat they contain, which are called lipomatous tumors (>70% of fat), myomatous (<10%), and angiomatous and mixed variants [1, 2, 5]. The immunohistochemical study of AML is always positive for HMB45 and frequent for S100 and actin [2, 4, 6]. The AML has always been regarded as benign tumor, with a slow growth and with no chances of a malign transformation [3, 4], but four works published from 2000 to now report the occurrence of 4 malignant AML cases that have or can develop the capacity to relapse and lead to vascular invasion [2–4].

The combined use of abdominal ultrasonography, CT, and MRI has increased preoperative diagnostic certainty of AML, especially in those cases when a fat component and some central and prominent abnormal vessels can clearly be detected; however the correct preoperative diagnosis does not exceed 50% [2–5]. The ultrasonography reveals a heterogeneous hyperechoic mass that sometimes is difficult to distinguish from a hemangioma [2, 4, 5]. The CT shows an AML with two parts: a peripheral angiomatous component and a lipomatous one with a low attenuation [1, 6]. The MRI of the AML reveals an intense signal in T1 and T2, and it seems that a somewhat higher specificity is obtained when compared with other imaging systems [1–6]. A differential diagnosis is suggested in the event of hepatocarcinoma and other liver tumors that may include a variable fat content (adenoma, lipoma, liposarcoma, sarcoma, GIST, metastasis, etc.) [1–3, 6]. The FNAP technique may yield a low rate of correct diagnoses, so its utility is reduced [1, 4].

AML is generally asymptomatic, but big-sized tumors may produce compressive symptoms, such as abdominal pain in superior hemiabdomen, plenitude sensation after intake of food, palpable mass, and other symptoms that include weight loss or fever [1–4, 6]. There seems to be a correlation between a higher size than 5 cm and the occurrence of symptoms [4].

General indications for the resection of benign liver tumor (BLT) include diagnostic doubt, occurrence of symptoms, or onset of complications [8, 10]. BLT exeresis represents 10% of the whole number of liver resections [8]. There is currently a tendency to perform these resections by laparoscopy when it is possible, although this laparoscopic approach should not increase the amount of surgery indications for BLT [10, 11].

Accepted indications for resection of AML include symptomatic patients, those cases where malignancy cannot be excluded, rapid growth tumors, and lesions with an exophytic component, as this last increases the risk of rupture [6]. Some authors recommend the resection of all AMLs larger than 5 cm [3, 4]. The most accepted criteria for a preserving management comprise AML cases that are smaller than 5 cm, asymptomatic, whenever their histology has been tested through FNAP, and uninfected by hepatotropic viruses, as it could lead to an erroneous diagnosis of hepatocarcinoma [3, 4]. The above-mentioned existence of malignant cases has reopened the debate on the need to approach all AML cases. The only medical available treatment for AML—although no randomized trial has been carried out yet—is based on sirolimus, as it seems to reduce the AML size by inhibiting mTORC1 [7]. Our opinion is that asymptomatic AML, less than 5 cm, in a noncirrhotic liver could be observed but after explaining to the patient the very low (3%) but possible incidence of cancer. The rest of the AML, symptomatic, quick growing, bigger than 5 cm, not of clear diagnosis, should be resected.

The first laparoscopic resection of the liver (LRL) was performed in 1992, but it had no exponential growth until the last five years when it coincided with a better technology that did not exist previously [8, 12]. The more frequent LRL (65%) comprises minor hepatectomies (left lateral sectionectomy and atypical resections), and segments II to VI are regarded as the ideal ones for LRL [8, 9, 12]. A progressive experience on LRL has made possible a higher number of resections on segments that initially were more complex and major hepatectomies [8, 9, 12]. 

RLR on BLT is an excellent indication since it rules out the risk of tumor dissemination, presents the benefits of the laparoscopic surgery for usually young patients, and reduces the mean in-hospital stay and recovery time [8, 9, 12]. The potential disadvantages of LRL include slow progress of the learning curve, bleeding, inaccurate assessment of lesions, which may go unnoticed, and the risk of air embolism [12]. In 2009, an exhaustive worldwide revision was published on 2801 patients who had undergone LRL, out of which 44.7% (*n*  =  1253) of LRL procedures dealt with BLT cases [12]. There are, though, few publications devoted exclusively to the implementation of LRL on BLT cases [8, 9]; the two most numerous series on this issue totalized 70 patients and only two of them belonged to the AML group [8, 9]. The percentage of complications concerning LRL on BLT varies between 10 and 20%, while mortality is 0%, a fact that proves that LRL is feasible and safe [8, 9].

The classical LRL technique employs 5 trocars (two of 12 mm, one of 10 mm, and two of 5 mm) and systematic portal clamping [8, 9]. However, the published series reveal that in a percentage of patients, which varies between 12.5% and 46%, the technique was performed without Pringle's maneuver. Our posture is that in peripheral resections concerning small tumors, or in segmentectomies, it is not so strictly necessary to perform the portal clamping, although it is advisable to be prepared for any contingency. There is only one publication that mentions the use of three trocars in 9 LRL cases [13]. All patients had malign tumors (8 hepatocarcinomas and one hepatic metastasis), superficially located from segments II to VI and VIII and exhibited a mean size of 3 cm. Resection was performed with ultrasonic scalpel. The mean time of procedures was 2 hours, Pringle's maneuver was not used, blood loss amounted to 75 mL, and morbidity-mortality was negative [13]. Our opinion is that in selected patients those peripheral lesions smaller than 5 cm can be resected with only three trocars instead of the classical 5 ones. 

The SILS technique is another option for these minor resections. The main problems for liver SILS surgery are new learning curve, loss of instrumental triangulation, vision problems because camera and instruments are parallel, expensive costs and incisional hernias [14–19]. After an extensive bibliographical search, only 9 liver SILS surgeries have been found, two laparoscopic fenestration of simple cyst and 7 liver resections. Lesions were always located in left lateral segment [14–19]. In every case, the surgeon's opinion is that liver SILS surgery is complex, technically demanding, and not always feasible. Besides, in some cases additional ports are required [18, 19]. The advantages of 3 trocars technique versus SILS are conventional devices, more anatomical vision, nonlearning curve, and costs.

So, three-port laparoscopic resection is safe and feasible in some liver lesions. Pringle's maneuver should be prepared but frequently is not necessary to use. AML is a rare neoplasm of the liver with not well-defined malignant potential so surgical resection in suitable patients is indicated.
